# Anticoagulant Activity and Structural Characterization of Polysaccharide from Abalone (*Haliotis discus hannai* Ino) Gonad

**DOI:** 10.3390/molecules21060697

**Published:** 2016-06-08

**Authors:** Jun Zhao, Jingfeng Yang, Shuang Song, Dayong Zhou, Weizhou Qiao, Ce Zhu, Shuyin Liu, Beiwei Zhu

**Affiliations:** 1 School of Light Industry and Chemical Engineering, Dalian Polytechnic University, Dalian 116034, China; kittyufo@163.com; 2 School of Food Science and Technology, National Engineering Research Center of Seafood, National and Local Joint Engineering Laboratory for Marine Bioactive Polysaccharide Development and Application, Dalian Polytechnic University, Dalian 116034, China; yangjf@dlpu.edu.cn (J.Y.); songs1008@163.com (S.S.); zdyzf1@163.com (D.Z.); silvia8801@163.com (C.Z.); 18340801443@163.com (S.L.); 3 Clinical Laboratory, Dalian Municipal Central Hospital Affiliated of Dalian Medical University, Dalian 116033, China; qiaoweizhouzxyy@163.com

**Keywords:** polysaccharide, *Haliotis discus hannai*, APTT, TT, PT, backbone

## Abstract

In this study, we aimed at characterizing the structure and the anticoagulant activity of a polysaccharide fraction (AGP33) isolated from the gonads of *Haliotis discus hannai* Ino. AGP33 was extracted by enzymatic hydrolysis and purified by ion-exchange and gel-filtration chromatography. The backbone fraction of AGP33 (BAGP33), which appeared to contain of mannose, glucose and galactose, was prepared by partial acid hydrolysis. According to methylation and nuclear magnetic resonance (NMR) spectroscopy, the backbone of AGP33 was identified as mainly consisting of 1→3-linked, 1→4-linked, and 1→6-linked monosaccharides. AGP33 is a sulfated polysaccharide with sulfates occur at 3-*O*- and 4-*O*-positions. It prolonged thromboplastin time (APTT), thrombin time (TT) and prothrombin time (PT) compared to a saline control solution in a dosage-dependent manner. AGP33 exhibited an extension (*p* < 0.01) of APTT compared to the saline group at concentrations higher than 5 μg/mL. AGP33 exhibited higher anticoagulant activity than its desulfated product (AGP33-des) and BAGP33. The results showed that polysaccharide with higher molecular weight and sulfate content demonstrated greater anticoagulant activity.

## 1. Introduction

Abalone, a very large edible sea snail, is a marine gastropod mollusc which belongs to the Haliotidae family. *Haliotis discus hannai* Ino has been regarded as a functional food since ancient times in China, and now the aquacultural production of this species reached 115,397 ton in 2014 [1] However, the gonads, which account for one-third of the total mass of abalone pleopod, are routinely discarded during processing. The disposal of the gonad not only leads to underutilization of valuable biomass, but also creates an environmental challenge of how to handle such a large quantity of biowastes.

An earlier study has shown that polysaccharides from abalone gonads were sulfated polymers accounting for one-tenth of the total gonad mass [2]. It is widely recognized that most of the sulfated polysaccharides can serve as anticoagulants [3,4,5,6]. For example, the potent anticoagulant heparin can activate antithrombin III, which blocks thrombin from clotting the blood stream. Heparin has been used *in vivo* and *in vitro* for clinical application in thrombotic desease [7]. However, the availability of heparin seems to be somewhat limited by its low concentration in porcine intestine or bovine lung, which are its main natural sources. Furthermore, the incidence of prion-related diseases in cows means that bovine heparin runs a risk of contamination which has to be carefully monitored. In the meantime, the ever-increasing demand for antithrombotic therapy calls for alternative sources of anticoagulant and antithrombotic compounds [8]. It has been shown that sulfated polysaccharides with anticoagulant activity are most abundant in marine invertebrates and marine algae [9,10]. Hence, abalone gonads could eventually become a valuable source of polysaccharides with anticoagulant properties.

In this study, we aimed at characterizing the structure and the anticoagulant activity of a polysaccharide fraction (AGP33) isolated from the gonads of abalone, whose potential can lead to a more effective commercial development of this natural product.

## 2. Results and Discussion

### 2.1. Preparation and Characterization of Abalone Gonad Polysaccharides

**Figure 1:**
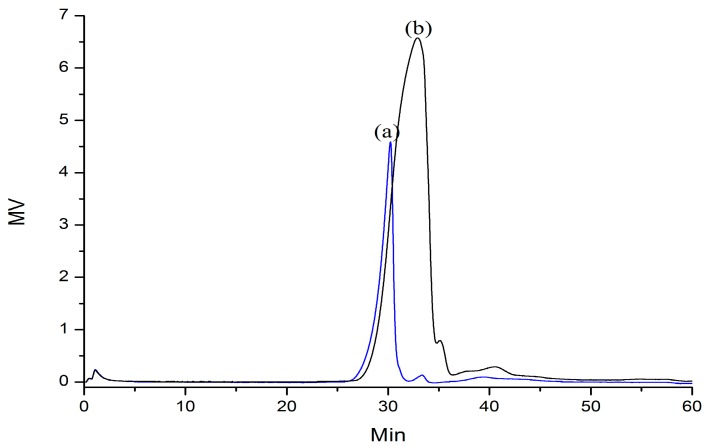
Elution profile of AGP33 (**a**) and its hydrolysis product BAGP33 (**b**) on SEC chromatography using a differential refraction detector.

The crude abalone gonad polysaccharides were fractionated by a column of DEAE-52 cellulose, yielding three fractions. The fraction obtained in 0.5 M sodium chloride was collected and further purified on a Sepharose CL-6B column to yield three sub-fractions as described by Yang *et al*. [2]. The third fraction (AGP33) eluted from Sepharose CL-6B has shown to be homogeneous by liquid chromatogrphy (peak (a), Figure 1). AGP33, with an average molecular weight of 27.5 kg/mol, contains 7.49% ± 0.34% sulfate groups, and was composed of mannose, rhamnose, glucuronic acid, glucose, galactose, xylose, arabinose, and fucose. The optical rotation of AGP33 was found to be ${\left[\alpha \right]}_{589}^{25{}^{\circ}C}$ = +103.3 which allowed the confirmation of the homogeneity of this fraction.

### 2.2. Partial Acid Hydrolysis of AGP33

BAGP33, the partial acid hydrolysis product of AGP33, appeared as a single symmetrical peak in its size exclusion chromatogram, suggesting that it was in its pure form. As shown in Figure 1, peak (b), the retention time of BAGP33 was a bit longer than that of AGP33, suggesting a possible degradation reaction caused a decrease of the molecular weight, indicating that the backbone of AGP33 was maintained in BAGP33, while the branches were cleaved after hydrolysis. BAGP33, with an average molecular weight of 17.0 kg/mol, contains 1.71% ± 0.03% sulfate groups. The monosaccharide composition of BAGP 33 appeared to be mannose, glucose and galactose, which were also the building blocks of AGP33.

### 2.3. Methylation Analysis of BAGP33

BAGP33 was then fully methylated and hydrolyzed with trifluoroacetic acid (TFA). It was converted into alditol acetates, and analyzed by GC-MS. As summarized in Table 1, the types of linkage of the monomers were identified. BAGP33 exhibited five types of monosaccharide linkages, including 1→3-linked α-d-glucose, 1→4-linked α-d-galactose, and 1→6-linked α-d-glucose, mannose and galactose. The lack of branched linkages in BAGP33 indicated that BAGP33 was the backbone of AGP33, since most the side chains of AGP33 were removed during acidic hydrolysis.

### 2.4. ^13^C- and ^1^H-NMR Analysis of AGP33

**Figure 2:**
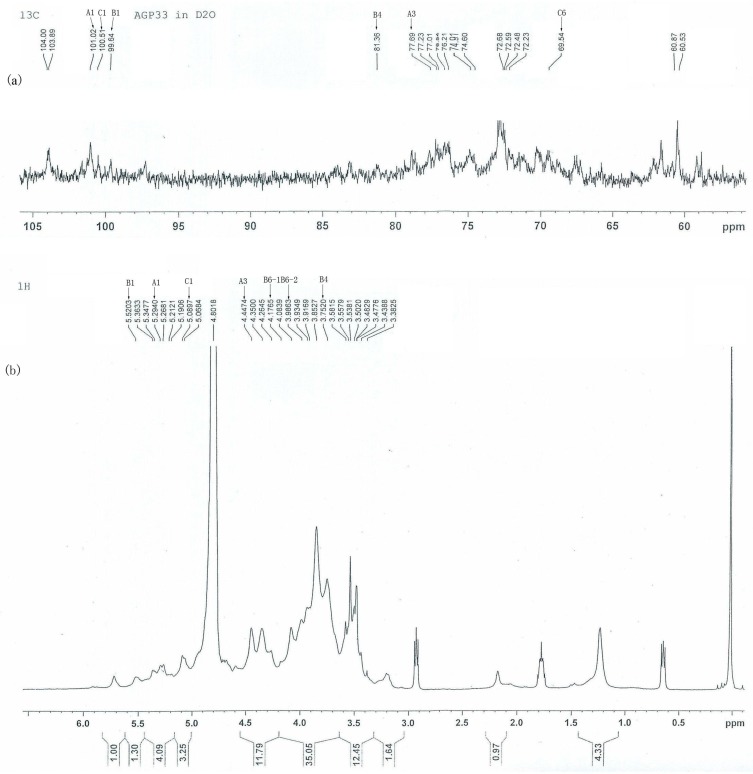
^13^C-NMR (**a**) and ^1^H-NMR (**b**) spectrum of AGP33. Residues A: 1,3-d-Glc*p*, Residues B: 1,4-d-Gal*p* and Residues C: 1,6-d-Glc*p*.

The ^13^C-NMR and ^1^H-NMR spectra of AGP33 are shown in Figure 2. The ^1^H-NMR spectrum shows strong anomeric H-1 (δ 5.52, 5.29, 5.09 and 5.07 ppm) proton signals, indicating the α-configuration of AGP33 monomers [11]. As shown in the ^13^C-NMR spectrum of AGP33, the resonances in the region of δ 97–101 ppm for C-1 can be attributed to the anomeric carbon atoms of pyranose, as the furan ring signals can be observed around δ 107–109 ppm [12]. Peaks presented at δ 101.02, 100.51, and 99.64 ppm correspond to C-1 of the 1→3-α-d-Glc*p*, 1→6-α-d-Glc*p*, and 1→4-α-d-Gal*p* residues, respectively. All the carbon signals indicate that they are all α-anomeric configuration [13]. The carbon signal correlated with the resonances at δ 69.54 ppm can be assigned to C-6 of the 1→6-α-d-Glc*p*, which is shifted about 9 ppm downfield compared to the resonance of standard methyl glycoside due to the effect of glycosylation [14]. Similarly, the signals at δ 77.69 and 81.36 ppm are assigned to C-3 of 1→3-d-Glc*p* and C-4 of 1→4-d-Gal*p*, respectively. Due to the fact that BAGP33 is the backbone of AGP33, the NMR spectra signal of BAGP33 is essentially in accord with the signal of AGP33. The AGP33 signals in the ^13^C-NMR spectrum are summarized in Table 1, which are in good agreement with the BAGP33 methylation results. Other signals in the ^13^C-NMR and ^1^H-NMR spectra (Table 1) are assigned based on their correlation with correlated spectroscopy (^1^H-^1^H COSY) and heteronuclear single-quantum coherence (HSQC) experiments [6,12].

### 2.5. HSQC Analysis of AGP33

**Figure 3:**
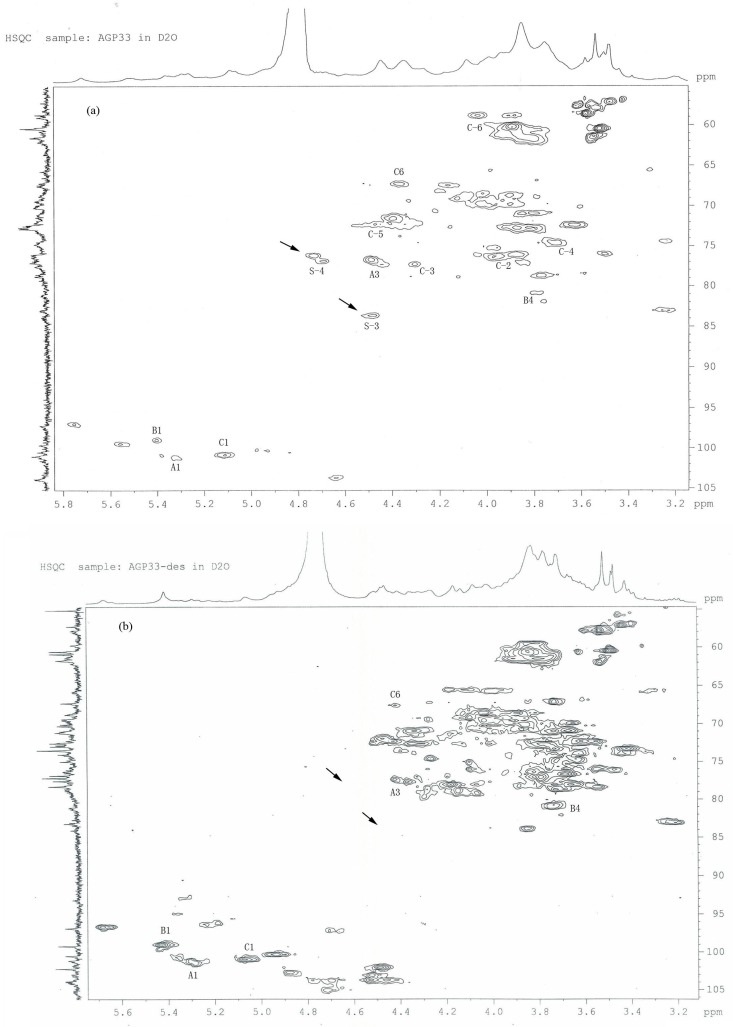
(**a**) HSQC spectrum of AGP33; (**b**) HSQC spectrum of AGP33-des (AGP33 without the sulfate group). The cross-peaks were assigned to 1,3-d-Glc*p* (residues A), 1,4-d-Gal*p* (residues B) and 1,6-d-Glc*p* (residues C). S-4: sulfate group in 4-*O*-position; S-3: sulfate group in 3-*O*-position.

The one-bond correlations of the ^13^C- and ^1^H-NMR spectra (Figure 3a) showed three main cross peaks in their anomeric regions. The C-1 signals at δ_C-1_ 101.02 and δ_C-1_ 99.64, assigned to the 1,3-d-Glc*p* (residue A) and 1,4-d-Gal*p* (residues B), showed cross-peaks with the H-1 resonance peaks at δ_H-1_ 5.29 and δ_H-1_ 5.52. The H-1 peak at δ_H-1_ 5.09 was assigned to 1,6-d-Glc*p* (residues C), since it correlated to the C-1 peak at δ_C-1_ 100.51. The cross peak between C-4 at δ_C-4_ 81.36 and the proton resonance at δ_H-4_ 3.75 allowed it to be assigned to the H-4-C-4 resonance of 1,4-d-Gal*p*. The cross peak between C-3 at δ_C-3_ 77.69 and the proton resonance at δ_H-3_ 4.45 allowed it to be assigned to the H-3-C-3 resonance of 1,3-d-Glc*p*. The H-6 resonance peaks appeared as doublets due to their coupling to each other. The C-6 peaks of 1,6-d-Glc*p* at δ 69.54 correlated well with the resonance peaks at δ_H-4_ 3.98 and 4.45. Some correlations between protons and their directly bonded carbons in 1,6-d-Gal*p* and 1,6-d-Man*p* were also observed in the HSQC spectrum, and these signals were useful for the assignment of their ^1^H and ^13^C resonances (Table 1).

### 2.6. ^1^H-^1^H COSY Analysis of AGP33

**Figure 4:**
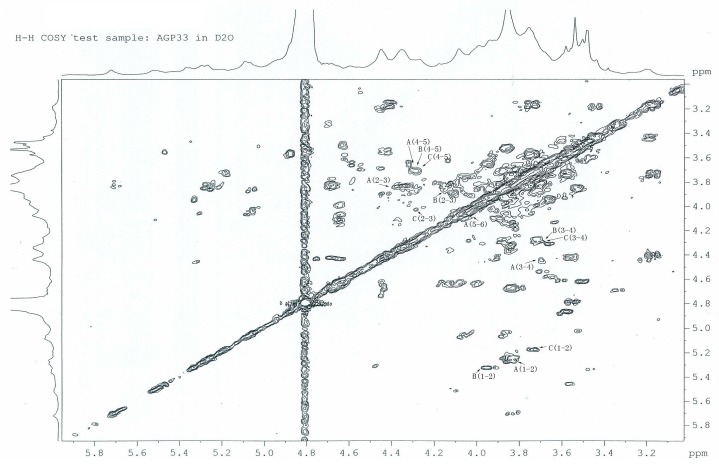
H, H-COSY spectrum of AGP33. The cross-peaks were showed the correlate of two adjacent hydrogen atom of each residue in which 1,3-d-Glc*p*, 1,4-d-Gal*p* and 1,6-d-Glc*p* referred to residues A, B and C respectively.

From the COSY spectrum shown in Figure 4, it was possible to correlate the H-1 of residue A (δ_H-1_ 5.29) with H-2 (δ_H-2_ 3.92). The H-1 of residue B (δ_H-1_ 5.52) correlates well with H-2 (δ_H-2_ 3.92). The H-1 of the 1, 6-d-Glc*p*, residues C (δ_H-1_ 5.09) correlates well with H-2 (δ_H-2_ 3.92). The corresponding C-2 and C-3 were also assigned in the HSQC spectrum. The peaks of H-2, H-3, C-2 and C-3 of the remaining residues (Table 1) were assigned by similar procedures.

### 2.7. Heteronuclear Multiple-Bond Correlation (^1^H- ^13^C-HMBC) Analysis of AGP33

**Figure 5:**
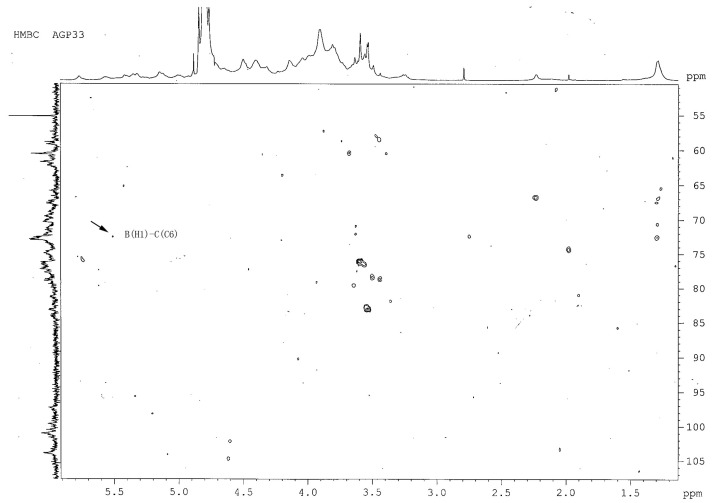
^1^H- ^13^C-HMBC spectrum of AGP33. The cross-peaks demonstrate that 1,4-d-Gal*p* (residue B) links directly to 1,6-d-Glc*p* (residue C).

The C resonance peaks of 1,3-d-Glc*p* (residue A), 1,4-d-Gal*p* (residue B) and 1,6-d-Glc*p* (residue C) were assigned by the analysis of the HMBC spectrum (Figure 5). In the HMBC spectrum, cross peaks H-1-C-6 (linkages of residue C, δ_C-6_ 69.54 to residues B, δ_H-1_ 5.52) are identified. This result suggests that the H-1 of 1,4-d-Gal*p* is linked directly to C-6 of the 1,6-d-Glc*p* residue. The cross peak at δ_H-1_ 5.52 and the resonances at δ_C-6_ 69.54 in HMBC spectrum indicate that 1,4-d-Gal*p* residues and 1,6-d-Glc*p* residues are also linked directly, constituting a disaccharide of the backbone of AGP33.

Based on the experimental data above, we deduced that the backbone of AGP33 may be consisted of (1→4)-linked galactose and (1→6)-linked glucose, linked together as a disaccharide unit of [→4)-α-Gal*p*-(1→6)-α-Glc*p*-(1→]_n_.

### 2.8. Analysis of the Position of the Sulfate Groups in AGP33

After the sample of AGP33 was desulfated (AGP33-des), the sulfate group position in AGP33 was detected by HSQC spectroscopy. The HSQC spectrum of AGP33-des was similar to that of AGP33 except for the two cross-peaks related to the sulfate groups, which disappeared in AGP33-des (Figure 3b). The cross peak at resonances δ_H-1_ 4.45 and δ_C-6_ 84.01 in HSQC spectrum was identified as a signal of a sulfate group acting on its adjacent 3-*O*-position atom in the monosaccharide (Figure 3a). Similarly, the cross peak at resonances δ_H-1_ 4.72 and δ_C-6_ 77.69 was identified as a signal of a sulfate group acting on its adjacent 4-*O*-position atom in monosaccharide (Figure 3a). The disappearance of the two cross-peaks in AGP33-des was attributed to the removal of the sulfate groups from their original position. Based on the above results, we suggest that sulfation of AGP33 might occur at 3-*O*- and 4-*O*-positions.

### 2.9. Anticoagulant Activity

**Figure 6:**
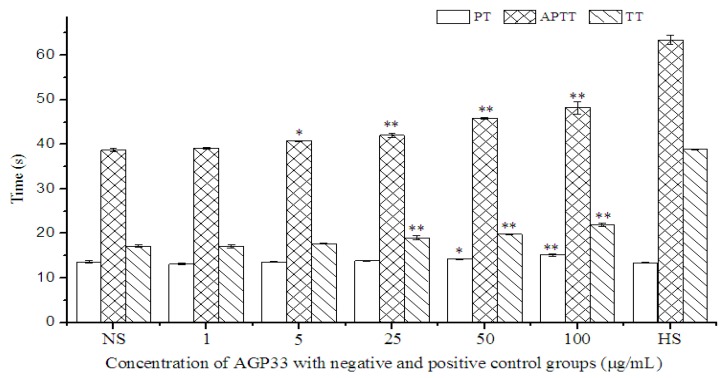
AGP33 prolonged APTT, PT and TT compared to normal saline (NS) group as a dose-dependent manner. The “*” means different to NS group (*p* < 0.05) and “**” means significant different to NS group (*p* < 0.01). HS was set as the positive control with concentration of 0.5 μg/mL.

The anticoagulant activity of AGP33 was evaluated by the classical coagulation assays for activated partial thromboplastin time (APTT), thrombin time (TT) and prothrombin time (PT), with normal saline solution (NS, 0.9%, *w/v*) as the negative control and heparin sodium (HS, 0.5 μg/mL) as the positive control. As shown in Figure 6, AGP33 extended APTT, PT and TT in a dosage-dependent manner. AGP33 exhibited a significant extension (*p* < 0.01) in TT, compared to the negative control, when its concentration was higher than 25 μg/mL. Transformation of fibrinogen into fibrin is known to be the last step in coagulation, and TT is a key indicator of this process. Hence, AGP33 might inhibit the conversion of fibrinogen into fibrin [6]. APTT was also extended significantly (*p* < 0.01) when AGP33 concentration was higher than 25 μg/mL when compared to the negative control. APTT is used to evaluate the coagulation factors such as VIII, IX, X, XII and prekallikrein in the intrinsic blood coagulation pathway [15]. These results confirmed that AGP33 may interfere with the coagulation factors (VIII, IX, X, XII) during the intrinsic coagulation process. PT was extended significantly (*p* < 0.01) when AGP33 concentration was higher than 100 μg/mL. As PT is used to characterize the extrinsic coagulation factors, these results obtained for the anticoagulant activity suggest that AGP33 may inhibit both the intrinsic and the common coagulation pathways.

**Figure 7:**
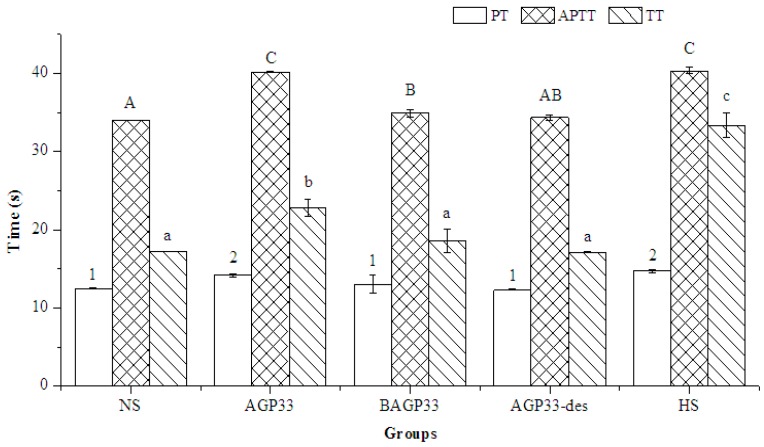
The anticoagulant activity of AGP33 compared with AGP33-des and BAGP33 at concentration of 50 μg/mL. NS means normal saline group. HS was a positive control with concentration of 0.5 μg/mL. Results are presented as means ± standard deviations (*n* = 3). Significant differences between different groups were evaluated by One-Way ANOVA (*post hoc* test: S-N-K). Values in the same dose of different groups with different numbers (1–2), lower case letters (a–c) and capital letters (A–C) are significantly different at *p* < 0.05.

The anticoagulant activity of AGP33-des and BAGP33 were tested. All the samples prolonged APTT in the concentration of 50 μg/mL, as shown in Figure 7. BAGP33 and AGP33-des showed differences from the blank control NS group in APTT but they could not prolong PT and TT. It was stated that the anticoagulant activity is associated to the high sulfate content of polysaccharides [16]. The low anticoagulant activity of AGP33-des supported this opinion. BAGP33 is the major fraction of AGP33 with little anticoagulant activity. This result suggested that both the molecular weight and degree of sulfation have essential influences on the anticoagulant activity of polysaccharides. Furthermore, the anticoagulant properties of the polysaccharides do not depend only on the percentage of sulphate residues, but rather, mostly on the distribution or position of sulphate groups and probably on the configuration of the polymer chains [9].

Previous studies indicated that there are many factors that affect the biological activity of polysaccharides, not only the structure, the configuration of the polymer molecules, the concentration of sulfate [9,10], molecular weight and branching structures [17], but also the position of sulfate groups, since the interaction of sulfated polysaccharides with coagulation cofactors and their target protease are specific [18]. There is an argument about how the sulfation pattern influences the anticoagulant activity of sulfated polysaccharides. The anticoagulant activity of sulfated cellulose significantly increased with the content of sulfate in the C-2 and C-3 sites, but not in the C-6 site [18]. On the other hand, a study on the anticoagulant properties of citrus pectin showed that the polysaccharide became more potent with the sulfation at C-6 [3]. AGP33 is a polysaccharide with sulfate substitutents at the 3-*O*- and 4-*O*-position. Although AGP33 can extend APTT and exhibited an extension (*p* < 0.05) compared to the negative control with concentration of 5 μg/mL, the polysaccharide of CP-CR_4_S sulfated at the 6-*O*-position showed a doubling of APTT at a concentration of 4.9 μg/mL [3]. AGP33 with sulfate at the 3-*O*- and 4-*O*-position has less potency compared to CP-CR_4_S sulfated at the 6-*O*-position at the same concentration. The absence of sulfate at the 6-*O*-position in AGP33 may explain its low anticoagulant activity. Therefore, the distribution of sulfate at the 6-*O*-position may be one of the crucial factors for the anticoagulant activity of the polysaccharides.

## 3. Experimental Section

### 3.1. Materials and Reagents

Gonads of abalone (*H.* *discus* *hannai* Ino) were obtained from Dalian Zhangzidao Group Co., Dalian, China. The fresh gonads were freeze-dried and smashed before extraction. Trifluoroacetic acid, monosaccharide standards, dextran molecular weight standards and MD-25 dialysis tubes (MWCO 7 kg/mol) were purchased from Sigma-Aldrich (St. Louis, MO, USA). Cellulose DEAE-52 and Sepharose CL-6B were purchased from Amersham Co. (Uppsala, Sweden). Activated partial thromboplastin time reagent (APTT, ellagic and bovine phospholipid), CaCl_2_ solution, prothrombin time (PT) reagent and thrombin time (TT) reagents were purchased from Stago Co. (Paris, France). All chemicals used in this study were of analytical reagent grade or above.

### 3.2. General Methods

The NMR spectra were recorded in D_2_O on an AV-500 spectrometer (Bruker, Bremen, Germany) operating at 500.13 and 125.75 MHz, respectively. The polysaccharide was dissolved in D_2_O, and chemical shifts were referenced with Me_4_Si. Sulfate content was assessed by barium chloride method using potassium sulfate as the standard [19]. Desulfation of polysaccharide was carried out using the pyridinium salts [20]. The sulfate group positions in the polysaccharide were revealed by HSQC spectroscopy (Bruker). All chromatographic assays were conducted with the phenol-sulfuric acid detection method [21].

### 3.3. Isolation, Purification and Molecular Weight Determination of Abalone Gonad Polysaccharide

The isolation and purification of the polysaccharide fraction AGP33 was conducted as previously reported [2]. The homogeneity of AGP33 was determined using a Waters e2695 liquid chromatography system (Waters, Milford, MA, USA) equipped with a TSK-gel G4000PWXL (TOSOH, Tokyo, Japan) column (7.8 mm × 300 mm) and a differential refraction detector of 2414 (Waters). Ten μL of sample solution (1 mg/mL) was analyzed in each run, with distilled water as the eluent at a flow rate of 0.2 mL/min. The molecular weights of samples were determined by a column of Sepharose CL-6B (1.6 cm × 80 cm), eluted with 0.15 M NaCl at a flow rate of 15 mL/h. The column was calibrated by dextran standards (6.7 × 10^5^ g/mol, 4.1 × 10^5^ g/mol, 2.7 × 10^5^ g/mol, 8 × 10^4^ g/mol, 1.2 × 10^4^ g/mol, Sigma-Aldrich).

### 3.4. Monosaccharide Composition of AGP33

The monosaccharide composition of AGP33 was assessed by submitting their acetylated aldononitrile derivatives to gas chromatography, according to Rajendra *et al*. with some modifications [22]. Briefly, AGP33 (10 mg) was hydrolyzed with 2.0 M TFA (2 mL) at 100 °C for 4 h, followed by evaporation with nitrogen gas to dryness. The residue was then mixed with hydroxylamine hydrochloride (8 mg), inositol (1 mg) and pyridine (1 mL), and incubated at 90 °C for 30 min. After allowing it to cool down to room temperature, the mixture was acetylated with acetic anhydride (0.5 mL) at 90 °C for 30 min. The alditol acetate was examined by gas chromatography (GC) on an Agilent 6890N system (Agilent, Palo Alto, CA, USA) equipped with a HP-5 column (0.32 mm × 30 m) and a flame-ionization detector. Nitrogen was used as the carrier gas (1 mL/min). The injector temperature was kept at 250 °C (split injection 20:1). The operation was performed at a column temperature programmed for 130 °C, holding for 5 min, then increasing to 200 °C at 3 °C/min, holding for 5 min and increasing to 240 °C at 3 °C/min, finally holding for 1 min at 240 °C.

### 3.5. Partial Acid Hydrolysis and Purification of Depolymerized AGP33

AGP33 (50 mg) was dissolved in 0.05 M TFA solution (2 mL) in a hydrolysis tube. After that, the tube was filled with pure nitrogen gas to replace the original air. The mixture was incubated at 95 °C for 3 h, centrifuged at 10,000 g for 3 min and then the supernatant was recovered. TFA in the supernatant was removed by evaporation and then the sample was purified by passing through a column of Sephadex G-75. The main polysaccharide fraction, named BAGP33, was recovered, dialyzed, and lyophilized for further analysis.

### 3.6. Methylation Analysis of BAGP33

BAGP33 was methylated according to the method of Needs and Selvendran [23]. The completion of methylation was evidenced by the disappearance of the hydroxyl absorption peak at 3400 cm^−1^ in the IR spectrum. The methylated product was depolymerized with 85% formic acid for 4 h (100 °C) and then further hydrolyzed with 2 M TFA for 6 h at the same temperature. The hydrolysates were then reduced and acetylated. The products were quantitatively analyzed by GC-MS method [24]. The exact types of methylated alditol acetates and their molar ratios were obtained.

### 3.7. Blood Coagulation Assays

APTT, TT and PT tests were used for the determination of the anticoagulant activity of the polysaccharide *in vitro*. Human blood samples were obtained from healthy donors in Dalian Central Hospital, Liaoning Province, China. Each sample was mixed with sodium citrate solution (3.8%, *w*/*w*) at a ratio of 9:1 (*v*/*v*). The mixture was centrifuged at 3000 g for 10 min to isolate the human plasma for further usage. The polysaccharide AGP33 was dissolved in saline solution (0.9%, *w/w*) to a final concentration of 1, 5, 25, 50 and 100 μg/mL, respectively, and mixed with human plasma to form solutions for the following anticoagulant evaluation. For APTT clotting assay, the mixture of plasma and AGP33 (0.1 mL) prepared as described above was mixed with 0.1 mL APTT reagent and incubated at 37 °C for 3 min. Pre-incubated 0.1 mL of 0.025 M CaCl_2_ solution was added and clotting time was recorded with an automated coagulometer (Stago-R, Pairs, France). In PT clotting measurement, the mixture of plasma and AGP33 (0.1 mL) prepared as described above was incubated at 37 °C for 2 min; then 0.2 mL of pre-incubated PT reagent was added, and the clotting time was recorded. For TT clotting test, the mixture of plasma and AGP33 (0.1 mL) prepared as described above was incubated at 37 °C for 2 min and 0.2 mL of pre-incubated TT reagent was added, and the clotting time was recorded. All assays were repeated three times and mean values calculated.

### 3.8. Statistical Analysis

All the tests were conducted with three replicates (*n* = 3). Data are presented as mean ± standard deviation (SD). Mean values were compared by One-factor Analysis of Variance (ANOVA) and the differences between means were evaluated by using S-N-K test as well as *t*-test. The statistical analysis was performed by using SPSS 16.0 software (SPSS Inc. Chicago, IL, USA). Comparisons that yielded *p* values < 0.05 were considered significant.

## 4. Conclusions

The sulfated polysaccharide fraction isolated from the gonads of abalone (AGP33) is composed of mannose, rhamnose, glucuronic acid, glucose, galactose, xylose, arabinose, and fucose with the sulfate groups occurring at the 3-*O*- and 4-*O*-positions. The main sugar residues of the backbone of AGP33 were found to be 1→3-linked glucose, 1→4-linked galatose, and 1→6-linked glucose. The anticoagulant activity results of AGP33, BAGP33 and AGP33-des showed that polysaccharide with higher molecular weight and sulfate content demonstrated greater anticoagulant activity.

## Figures and Tables

**Table 1:** Methylation analysis of *BAGP33* and the NMR spectrum signals assigned for *AGP33*.

Linkage Pattern		Methylation Analysis		Chemical Shifts (δ) for the Carbon and Hydrogen Atoms					
		Methylated Product	Molar Ratio	C1	C2	C3	C4	C5	C6
				H1	H2	H3	H4	H5	H6
**A**	→3)-α-d-Glc*p* (1→	1,3,5-Ac_3_-2,4,6-Me_3_-Glc	6.7	101.02	76.54	77.69	72.48	72.23	60.53
				5.29	3.92	4.45	3.75	4.35	3.58, 4.18
**B**	→4)-α-d-Gal*p* (1→	1,4,5-Ac_3_-2,3,6-Me_3_-Gal	13.3	99.64	76.54	77.23	81.36	72.23	60.53
				5.52	3.92	4.35	3.75	4.35	3.58, 4.18
**C**	→6)-α-d-Glc*p* (1→	1,5,6-Ac_3_-2,3,4-Me_3_-Glc	6.3	100.51	76.21	77.23	72.68	72.23	69.54
				5.09	3.92	4.35	3.75	4.35	3.98, 4.45
	→6)-α-d-Man*p* (1→	1,5,6-Ac_3_-2,3,4-Me_3_-Man	6.0	101.70	76.21	77.23	72.68	72.23	70.3
				5.09	3.92	4.35	3.75	4.35	3.93, 4.18
	→6)-α-d-Gal*p* (1→	1,5,6-Ac_3_-2,3,4-Me_3_-Gal	3.2	101.02	76.21	77.01	72.59	72.23	68.3
				5.07	3.92	4.35	3.75	4.35	4.08, 4.18
